# Quinoline Quest: Kynurenic Acid Strategies for Next-Generation Therapeutics via Rational Drug Design

**DOI:** 10.3390/ph18050607

**Published:** 2025-04-22

**Authors:** Masaru Tanaka, István Szatmári, László Vécsei

**Affiliations:** 1 Danube Neuroscience Research Laboratory, HUN-REN-SZTE Neuroscience Research Group, Hungarian Research Network, University of Szeged (HUN-REN-SZTE), Tisza Lajos krt. 113, H-6725 Szeged, Hungary; 2 Institute of Pharmaceutical Chemistry and HUN-REN–SZTE Stereochemistry Research Group, University of Szeged, Eötvös u. 6, H-6720 Szeged, Hungary; szatmari.istvan@szte.hu; 3 Department of Neurology, Albert Szent-Györgyi Medical School, University of Szeged, Semmelweis u. 6, H-6725 Szeged, Hungary

**Keywords:** kynurenic acid (KYNA), quinoline, tryptophan (Trp), neuroprotection, neuroinflammation, excitotoxicity, blood–brain barrier (BBB), pharmacokinetics, structure–activity relationships (SARs), drug design

## Abstract

Background: Quinoline-derived metabolites exhibit notable chemical complexity. What causes minor structural alterations to induce significant changes in disease outcomes? Historically, eclipsed by more straightforward scaffolds, these chemicals serve as a dynamic hub in tryptophan metabolism, linking immunomodulation, excitotoxicity, and cancer. However, many of these compounds struggle to cross the blood–brain barrier, and we still do not fully understand how certain structural changes affect their bioavailability or off-target effects. Thus, contemporary research highlights halogenation, esterification, and computational modeling to enhance structure–activity relationships. Summary: This narrative review emphasizes the integration of rational drug design, multi-target ligands, and prodrug methods in enhancing quinoline scaffolds. We explore each molecule’s therapeutic promise, refine each scaffold’s design, and develop each derivative to maximize clinical utility. Translating these laboratory findings into clinical practice, however, remains a formidable challenge. Conclusions: Through the synthesis of findings regarding NMDA receptor antagonism, improved oral bioavailability, and reduced metabolic instability, we demonstrate how single-site changes might modulate excitotoxicity and immunological signaling. Advancing quinoline-based medicines will yield significant advancements in neurology, psychiatry, and oncology. This enlarged framework fosters collaborative discovery, engages various audiences, and advances the field towards next-generation disease-modifying therapies. Robust preclinical validation, patient classification, and comprehensive toxicity evaluations are crucial stages for achieving these extensive endeavors and fostering future therapeutic discoveries globally.

## 1. Introduction

**Figure 1:**
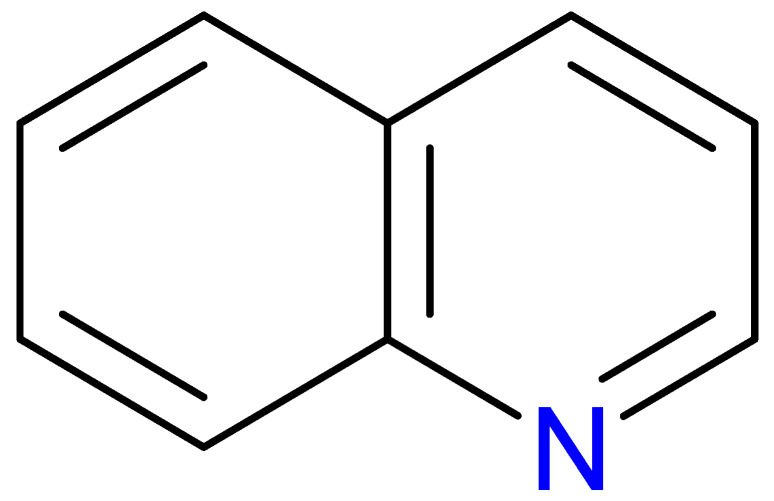
The structure of quinoline. The quinoline scaffold, featuring a fused benzene and pyridine ring, forms the structural backbone of many biologically active compounds.

Tryptophan (Trp) undergoes a fascinating series of biochemical transformations through kynurenine (KYN) metabolism that leads to the production of various neuroactive and immunomodulatory compounds [1]. At the heart of this metabolic arm lies a cascade of enzymes that generates an array of quinoline-based molecules, each with distinct and sometimes opposing effects on cellular and molecular processes (Figure 1) [2]. Among the most scrutinized products are kynurenic acid (KYNA) and quinolinic acid (QUIN), which play critical roles in modulating neurotransmission, safeguarding neurons, and orchestrating immune responses [3]. Under normal conditions, these molecules help maintain the delicate balance that protects and regulates neural function [4]. However, an overabundance or misregulation of these compounds can fuel diverse pathologies, spanning neurodegenerative conditions, psychiatric disorders, and even certain cancers [5]. Remarkably, these metabolites appear across many species and have preserved their functional importance for millions of years [6]. Their enduring presence suggests a fundamental role in maintaining physiological homeostasis, yet they can also become potent drivers of disease when their levels shift [7]. This duality underscores the importance of understanding both the biochemical nuances and the broader biological contexts in which these quinoline-based substances operate [3,8].

The quinoline skeleton lies at the heart of a wide variety of biologically active molecules, encompassing endogenous metabolites, such as KYNA and QUIN, as well as numerous synthetic derivatives found in pharmaceuticals and research tools [9]. Defined by a fused ring structure that merges a benzene ring with a pyridine ring, quinoline offers countless opportunities for chemical substitutions [10]. Small modifications—such as adding carboxyl or hydroxyl groups—can significantly change a molecule’s behavior. These changes affect solubility, stability, and even affinity for specific protein targets [11]. Halogenation, for instance, may boost lipophilicity and enhance central nervous system penetration, while the presence or absence of an acidic group can shape receptor-binding preferences [12]. In many cases, these structural nuances determine whether a particular quinoline derivative acts as a neuroprotective agent or exacerbates excitotoxic processes through receptors, like N-methyl-D-aspartate (NMDA) or G protein-coupled receptor 35 (GPR35) [13]. Endogenous metabolites derived from the Trp degradation route typically exhibit distinct features that help them coordinate intricate cellular signaling, whereas synthetic derivatives, exemplified by quinolone antibiotics, have been optimized for pathogen clearance and bioavailability [14]. Despite their diverse origins, each compound features the versatile quinoline core, underscoring the need for structural precision in creating targeted therapies [15].

A hallmark of these Trp-derived quinoline compounds is their profound influence on the delicate balance between neuroprotection and neurotoxicity [16]. Their functions are finely tuned by their relative concentrations, contextual factors, and micro-environment, shaping their cumulative effects [17]. Beyond their influence in the central nervous system, these metabolites also bridge metabolic and immune pathways, as illustrated by enzymes like indoleamine 2,3-dioxygenases (IDOs) and tryptophan 2,3-dioxygenase (TDO) [18]. When this biochemical system malfunctions, it can drive inflammation and alter disease pathways in autoimmunity and cancer [19]. Emerging evidence further suggests crosstalk with the aryl hydrocarbon receptor, hinting at broader physiological roles that extend beyond neuronal circuits [20]. Such multifaceted involvement underscores the need to appreciate how these molecules orchestrate, or disrupt, redox equilibrium, synaptic communication, and immune surveillance in tandem [21]. By influencing both protective and harmful pathways, these compounds form a paradox where even small imbalances can lead to disease [22,23]. Investigating this interplay is vital for deciphering the biology behind complex disorders, as well as for pinpointing novel therapeutic entry points grounded in the chemistry of quinoline-based metabolites.

In recent years, therapeutic strategies targeting Trp metabolism have drawn increased attention, supported by encouraging preclinical findings and a rise in clinical trials [24,25]. For instance, inhibitors of IDOs showed early promise in oncology by dampening immunosuppressive pathways and restoring anti-tumor responses [26]. However, some late-stage trials yielded disappointingly modest outcomes, illustrating the complexity of translating metabolic interventions into measurable clinical benefits [27]. Another approach uses KYNA analogs—such as 7-CKA—to help alleviate schizophrenia-like symptoms by modulating glutamate receptors [28]. Although these discoveries underscore the promise of quinoline-based therapeutics, many challenges still lie ahead [29]. Achieving adequate penetration across the blood–brain barrier (BBB) can be particularly daunting, as can mitigating off-target effects that may compromise patient safety [30]. Beyond endogenously derived agents, certain quinoline derivatives traditionally used for other indications, such as quinine and related molecules, are now being explored for their neuroprotective or anti-inflammatory properties [31]. Repurposing these compounds also accelerates clinical testing by leveraging existing safety and pharmacokinetic data [32]. Yet, optimizing dosage, delivery methods, and specificity remains a priority [33]. Moving forward, interdisciplinary teams can combine synthetic chemistry, pharmacology, and clinical insights to refine these agents and unlock their untapped therapeutic potential [34].

Despite expanding research on quinoline-containing molecules, critical questions remain unresolved [35]. One pivotal gap lies in our limited understanding of how specific structural modifications translate into particular biological effects, often termed structure–activity relationships [36]. Such insights are crucial for guiding rational drug design and for predicting how a new derivative might behave in living systems. Another hurdle is the fragmented nature of pharmacokinetic and safety data. Often, these findings come from small, isolated studies rather than comprehensive analyses [37]. This piecemeal approach complicates efforts to compare efficacy and toxicity across different quinoline scaffolds [37]. Furthermore, there is a noticeable lack of head-to-head evaluations between endogenous Trp-derived metabolites and synthetic or exogenous analogs, preventing a clear assessment of relative benefits and risks. Building on these concerns, the objectives of this review are threefold. The first is to systematically evaluate the wealth of endogenous and synthetic quinoline-based compounds for their therapeutic potential in various disease contexts. The second is to synthesize current knowledge about structure–activity relationships with the explicit goal of identifying a candidate inspired by KYNA that might serve as a leading drug template. Lastly, we aim to propose innovative strategies to address translational challenges, including targeted prodrug design and approaches that can engage multiple biological targets simultaneously.

## 2. Unveiling Endogenous Quinolines in the Kynurenine (KYN) Pathway: A Gateway to Kynurenic Acid (KYNA)-Driven Neuroprotection and Rational Drug Design

The metabolism of Trp produces a striking array of bioactive compounds [38]. KYNA, QUIN, nicotinic acid (NA), picolinic acid, xanthurenic acid (XA), and quinaldic acid each exert profound effects on human physiology [39]. Recent findings indicate that these metabolites serve as delicate modulators capable of shifting the balance between neuroprotection and neurotoxicity [40,41]. Recent studies indicate that KYNA’s protective profile extends to antioxidant mechanisms, beyond its well-characterized role in blocking excitotoxic glutamate receptors [42]. By directly scavenging reactive oxygen species and enhancing enzymatic antioxidant systems—such as superoxide dismutase and glutathione peroxidase—KYNA can mitigate oxidative stress in both neuronal and peripheral tissues [43]. These antioxidant properties are especially relevant in neurodegenerative disorders where imbalances in redox homeostasis fuel disease progression [44]. Integrating these newer findings with KYNA’s established immunomodulatory and neuroprotective roles further underscores its multifaceted potential as a therapeutic agent [45]. KYNA, often described as a safeguard against excitotoxicity, dampens glutamatergic signaling [46]. In contrast, QUIN can escalate excitatory activity to dangerous extremes, contributing to neuronal injury under pathological conditions [47]. NA supports redox homeostasis, while picolinic acid and XA coordinate metal ions, subtly influencing cellular stability [48]. Quinaldic acid, a relatively understudied molecule in this network, has roles that are only now beginning to emerge [49]. These metabolites do not function in isolation. They respond to the surrounding microenvironment and reshape it in ways that may ultimately define the trajectory of health and disease [50] (Table 1).

### 2.1. Structural and Functional Synergy

The quinoline core appears in a range of Trp-derived metabolites, revealing an intriguing contrast in their biological effects [2]. KYNA and QUIN emerge from the same metabolic pathway, yet they are traditionally considered to operate on opposite ends of the spectrum [75]. One protects neurons by dampening excessive excitatory signaling; the other drives excitotoxic damage often linked to inflammation [96]. This shared structural backbone, paired with such starkly different outcomes, suggests a delicate evolutionary balance shaped by minor molecular adjustments [2]. Consider how slight modifications to substituent groups on the quinoline ring can shift a molecule’s interaction with receptors, ion channels, or transporters [97]. Even fluctuations in pH or enzyme activity are enough to sway the system toward protection or harm [98]. There is more: metal ions and cofactors add another layer of control, either amplifying or suppressing activity [99]. The challenge now lies in identifying how to steer this balance [100]. Could targeted changes to the quinoline scaffold enhance KYNA’s protective roles or curb the destructive potential of QUIN [101]? Understanding these subtle structural cues opens the door to designing molecules with more favorable effects on neural health.

Small structural modifications within quinoline-based metabolites can significantly influence their biological activity and stability [102]. Altering the position of a hydroxyl group, as observed in XA, affects the hydrogen-bonding capacity and lipophilicity, which may shift receptor affinity and metabolic processing [103]. These effects are particularly relevant when examining interactions with glutamate receptors, such as NMDA, where even minor variations can determine whether a metabolite contributes to neuroprotection or excitotoxicity [104]. Similar patterns emerge with GPR35, a receptor that responds differently based on the specific substituents attached to the quinoline scaffold [105]. Variations in these groups regulate receptor engagement and downstream signaling pathways, shaping distinct physiological outcomes [98]. Alongside receptor interactions, metabolic stability is also sensitive to changes in ionization states and enzyme susceptibility, both of which are closely tied to the positioning of carboxyl, hydroxyl, or amino groups [106]. Mapping these structural features across endogenous metabolites provides insights into how they influence pathological processes [107,108]. Examining the relationship between the substitution patterns, receptor selectivity, and metabolic half-life may contribute to the design of compounds that adjust the activity of quinoline derivatives in targeted therapeutic contexts.

### 2.2. Key Gaps in Translation

Despite their diverse biological activities, translating endogenous quinoline-based compounds into therapeutic interventions faces significant hurdles [109]. A chief concern is poor bioavailability, particularly evident in KYNA [110]. Its polar carboxylate group hinders passive diffusion across biological membranes and limits the ability to reach target sites in sufficient concentrations [111]. On the other hand, picolinic acid, while exhibiting promising immunomodulatory and metal-chelating activities, undergoes rapid metabolism, leading to a short half-life and unstable plasma levels [112]. These kinetic and physicochemical drawbacks are further complicated by the complexity of enzymatic pathways involved, where small fluctuations in metabolic rates can greatly alter the overall balance of protective versus pathogenic metabolites [113]. Such pharmacokinetic challenges underscore the difficulty of achieving consistent dosing in clinical settings [114]. As highlighted in recent efforts to refine KYN system monitoring, relying solely on static concentrations or simplistic ratios may be insufficient. A more nuanced, integrative biomarker framework is needed to capture the dynamic regulation of tryptophan metabolism and guide precision medicine applications in neuropsychiatric and neurodegenerative disorders [115]. Moreover, existing animal models do not always recapitulate human physiology, complicating the interpretation of efficacy and toxicity data [62]. Without efficient strategies to enhance bioavailability—such as prodrug approaches, targeted delivery systems, or structural modifications to increase lipophilicity—these compounds may struggle to meet clinical requirements [116,117]. As research deepens our understanding of how structural and functional attributes influence pharmacokinetics, efforts to overcome these limitations can be directed more precisely, potentially unlocking the therapeutic promise that many of these metabolites have long suggested.

Translating endogenous quinoline compounds into real-world treatments often involves navigating paradoxical biological roles, as exemplified by NA [118,119]. Widely recognized for its capacity to modulate lipid metabolism and improve cardiovascular profiles, NA also harbors less-appreciated functions in inflammatory pathways [120]. This duality creates both opportunity and challenge: on one hand, its impact on lipid-lowering has made it a mainstay in certain therapeutic regimens; on the other hand, emerging studies suggest that NA can engage molecular cascades involved in neuroinflammation, potentially influencing conditions like neurodegenerative diseases [121,122]. Such conflicting actions underscore a larger theme in quinoline-related research, where a single molecule may oscillate between protective and pathogenic effects depending on the context and concentration [123]. The incomplete mapping of these dual functionalities hampers our ability to refine dosing strategies or identify the specific patient populations who might benefit the most [98]. Additionally, the diverse array of downstream metabolites and receptors further complicates the picture, making it difficult to anticipate long-term outcomes [124]. As a result, bridging the gap from bench to bedside requires a thorough understanding of how NA’s manifold roles intersect. Only by disentangling these intricate mechanisms can researchers devise targeted interventions that harness the therapeutic benefits of NA while minimizing unanticipated consequences.

A major obstacle in harnessing the therapeutic potential of endogenous quinoline compounds lies in their limited tissue penetration and rapid clearance [56]. KYNA, for example, is known to possess neuroprotective properties but struggles to traverse biological barriers effectively, largely due to its polar carboxyl group [110]. One promising strategy is to employ prodrug approaches, such as 4-chlorokynurenine and SZR-104, whereby this polar moiety is temporarily masked through esterification or similar chemical modifications [56,125]. By doing so, researchers can enhance lipophilicity, potentially improving permeability across the gut or BBB. Once inside target tissues, endogenous enzymes would cleave the ester bond, liberating active KYNA precisely where it is needed [126]. Another avenue involves synthesizing hybrid molecules, combining the core quinoline structure with lipid-soluble components [127]. This approach aims not only to improve membrane permeability but also to confer additional functional advantages, such as greater stability or targeted delivery [128]. For instance, merging KYNA with a fatty acid chain could optimize drug distribution in neuronal membranes, where excitotoxic processes frequently unfold [69]. While these strategies are conceptually appealing, they require systematic evaluations to ensure that altered pharmacokinetics do not come at the cost of diminished efficacy or unexpected side effects. Nevertheless, these innovations are promising for turning endogenous metabolites into practical therapies that address significant clinical needs.

One way to translate preclinical findings into clinical success is through dual-targeting compounds. These agents can influence key Trp-metabolizing enzymes while also modulating neuroinflammatory pathways [129]. By tackling both processes at once, these agents have the potential to dampen the production of harmful quinoline-based metabolites, such as QUIN, while also curbing inflammatory cascades that can exacerbate neuronal damage [130]. For example, inhibiting enzymes like IDOs or TDO while suppressing pro-inflammatory cytokines may reduce excitotoxic damage in neurodegenerative or chronic inflammatory conditions [131,132,133]. In diseases like multiple sclerosis, recent work emphasizes the critical importance of accurately monitoring redox dynamics as both a diagnostic tool and a therapeutic target, revealing that an oxidative imbalance directly influences disease progression and treatment responsiveness [134]. The rationale is to address multiple disease mechanisms in parallel, thereby enhancing therapeutic efficacy and possibly reducing the likelihood of resistance or relapse [135]. However, designing these dual-acting agents demands a nuanced understanding of each target’s regulatory networks, as altering enzyme activity may unintentionally shift the balance toward alternative metabolic routes or trigger compensatory immune responses [136]. Moreover, the BBB remains a formidable challenge, necessitating delivery strategies that ensure sufficient drug concentrations within the central nervous system [137].

While enhancing BBB penetration is essential, true therapeutic success often hinges on directing molecules to the specific CNS regions and cell populations most affected by disease. Recent work on ligand-decorated nanoparticles and receptor-specific peptides illustrates how researchers can guide drugs toward, for example, the hippocampus in Alzheimer’s disease or the substantia nigra in Parkinson’s, rather than distributing agents uniformly throughout the brain. Equally important is ensuring that active compounds localize to the relevant intracellular compartments—such as mitochondria or lysosomes—where key neuroprotective or anti-inflammatory mechanisms operate. By combining BBB-focused strategies with region- and cell-specific design, future quinoline-based therapies may significantly improve both efficacy and safety, reducing off-target burdens and optimizing outcomes in complex neurological conditions. Despite these hurdles, dual-targeting compounds offer a forward-thinking strategy, uniting key biochemical nodes into a single intervention that could revolutionize how we harness endogenous quinoline metabolites for clinical benefits.

## 3. Expanding the Quinoline Landscape: Derivatives Beyond the Kynurenine (KYN) Metabolic Pathway

Isonicotinic acid (INA) shares a structurally similar pyridine ring with KYNA’s quinoline core, creating promising opportunities for scaffold hybridization. These include investigating underexplored agents, like dipicolinic acid (DPA), in neurodegenerative applications [138]. However, these compounds may present limitations, including off-target bacterial DNA gyrase inhibition (e.g., nalidixic acid) or toxicity concerns (cinchoninic acid derivatives) [139,140]. Structural homology between isonicotinic acid (INA)’s pyridine ring and KYNA’s quinoline core highlights opportunities for scaffold hybridization and the exploration of underexamined agents, like dipicolinic acid (DPA), in neurodegeneration (Table 2). Further, systematic SAR studies on broad-spectrum drugs, exemplified by quinidine, could isolate neuroactive fragments for targeted therapeutic applications and ultimately safer use [141].

Despite the different historical trajectories of classical quinoline agents (like antimalarials and antibiotics) and those directly linked to the KYN pathway, they share structural underpinnings and overlapping challenges. Lessons from modifying older quinoline frameworks to improve safety, enhance stability, or extend clinical utility can be leveraged to optimize newer KYNA-inspired compounds. For instance, understanding how subtle ring substitutions in antibacterial quinolones influence enzyme targeting may inform strategies to fine-tune receptor specificity and pharmacokinetics in KYNA analogues. By connecting these two research streams, we establish a cohesive framework that underscores the universal importance of precise scaffold manipulation in drug discovery.

### 3.1. Repurposing Potential

XA, although not a central intermediate in the KYN pathway, illustrates how precise structural changes can influence receptor interactions and metabolic stability [210]. Introducing a hydroxyl group at specific sites on the quinoline ring modifies hydrogen bonding capacity, which may shift affinity toward NMDA receptors or GPR35 [105]. Such alterations affect not only receptor binding but also downstream processes, including neurotransmission and immune signaling [211]. Adjustments in stereochemistry or the addition of other substituents can further redirect molecular preferences, raising the possibility of selectively targeting distinct physiological pathways [212]. Yet, enhancing receptor specificity often comes with trade-offs [213]. For instance, while certain hydroxylations strengthen binding, they may also increase vulnerability to enzymatic degradation [214]. Strategies, such as incorporating protective groups or additional ring modifications, can help extend the plasma half-life and improve pharmacokinetic profiles [215]. Beyond the structural design, patient stratification based on genetic variations in enzymes, like IDOs and kynurenine 3-monooxygenase (KMO), offers a route to refine therapeutic applications [216]. Identifying metabolic subgroups may improve treatment outcomes and reduce adverse effects, particularly in neurodegenerative and neuroinflammatory conditions, where individual responses vary widely [217].

Quinoline derivatives beyond the KYN pathway present structural opportunities but are often limited by safety concerns that complicate therapeutic development [109]. Nalidixic acid, widely recognized for its antibacterial activity through the inhibition of DNA gyrase, raises concerns when considered for neuroprotective or immunomodulatory purposes [218]. Interference with DNA metabolism, while effective against bacterial targets, may introduce risks to human cells, particularly in long-term or systemic applications [219]. Similar challenges appear with cinchoninic acid derivatives, which share structural features with KYNs yet exhibit toxicity that restricts their clinical use [220]. In several cases, these effects appear to be linked to the formation of reactive metabolites or unintended interactions with metabolic enzymes [221]. Even minor modifications to the quinoline scaffold can redirect metabolic pathways, sometimes amplifying toxic outcomes [222]. Addressing these complexities requires a detailed understanding of structure–toxicity relationships and strategies that preserve pharmacological activity while minimizing off-target effects [223]. Approaches, such as selective functional group modifications and alternative synthetic routes, are under investigation [224]. Advancing these efforts depends on collaboration across medicinal chemistry, toxicology, and clinical research to reduce liabilities and support the development of safer quinoline-based compounds [225].

### 3.2. Bridging to Kynurenic Acid (KYNA)-Based Targets

Structural similarities between INA and KYNA have prompted interest in hybrid molecules that combine elements of both frameworks [226]. Each features a nitrogen-containing heterocycle capable of influencing receptor interactions and electron distribution [227]. By integrating portions of the quinoline core from KYNA with the pyridine ring of INA, researchers aim to enhance selectivity at targets, such as NMDA receptors and GPR35. This approach allows for adjustments in the ring orientation and substituent placement, offering opportunities to fine-tune receptor engagement while addressing challenges related to metabolic stability [228]. The isonicotinic scaffold, in particular, provides flexibility for introducing functional groups that may improve solubility or permeability [229]. While these modifications hold promise for minimizing off-target effects, the relationship between structural changes and pharmacological outcomes remains complex [230]. Balancing synthetic accessibility with therapeutic potential requires a careful evaluation of how hybrid designs alter binding properties and metabolic pathways. Drawing from the existing knowledge of INA derivatives, ongoing studies will assess whether these structural combinations can deliver improved performance while limiting adverse effects, especially in applications involving neuroprotective and immunomodulatory pathways.

DPA, a pyridine-2,6-dicarboxylic acid, remains relatively underexplored despite its capacity to bind metal ions commonly implicated in neurodegenerative diseases [231]. Through interactions with iron, zinc, and copper, DPA may contribute to regulating the metal balance, a process closely linked to oxidative stress and protein aggregation [232]. Preliminary findings suggest potential roles in modulating inflammatory responses and excitatory signaling, raising questions about its relevance to therapeutic strategies aimed at neuroprotection [233]. Structurally, the resemblance between DPA’s pyridine core and the quinoline ring of KYNA presents opportunities to combine metal-chelation with receptor targeting [234]. This dual functionality may prove valuable, particularly in disorders where oxidative damage and glutamatergic dysregulation intersect [235]. Parkinson’s disease and Alzheimer’s disease are two examples where such mechanisms converge, though much remains unknown regarding DPA’s pharmacological behavior [236]. Determining its metabolic stability, toxicity profile, and specific enzyme interactions will be essential for guiding further development [237]. By applying knowledge from KYNA analog research, it may be possible to design compounds that extend the benefits of metal-binding while refining receptor selectivity, supporting efforts to address complex neurodegenerative conditions [238].

### 3.3. Innovative Directions

Identifying neuroactive components within broad-spectrum quinoline derivatives offers a pathway to develop treatments with fewer adverse effects [12]. Quinidine, primarily recognized for its role in modulating cardiac ion channels, provides a useful example [225]. Through structure–activity relationship analyses, specific molecular features can be mapped to isolate those contributing to neurological benefits, such as limiting excitotoxicity, while reducing the risk of arrhythmias [239,240]. Computational docking and high-throughput screening techniques assist in pinpointing functional groups that influence ion channels within the central nervous system, rather than the heart [241]. These insights guide the design of analogues tailored to interact with neuronal targets, including channels and receptors implicated in neurodegenerative disorders [242]. Achieving this balance between the therapeutic effect and safety often relies on incremental structural adjustments—altering side chains, introducing new substituents, or modifying ring systems can significantly affect the binding behavior and metabolic stability [243]. While quinidine’s properties are well characterized, questions remain about how best to adapt its framework for neurological applications [244]. Ongoing work with related compounds may help define strategies to refine multi-purpose quinoline scaffolds and explore their potential in treating conditions, such as epilepsy, chronic pain, and neurodegeneration.

## 4. Exogenous Horizons: Synthetic Quinoline Scaffolds in Rational Drug Design

Driven by rational design approaches, synthetic modifications to quinoline-based scaffolds have enhanced both target engagement and pharmacokinetic profiles [245]. For example, chlorination in 7-CKA and sulfonamide substitution in Gavestinel improved NMDA receptor blockade and bioavailability, respectively [246,247,248,249]. Despite encouraging preclinical results, clinical outcomes have sometimes fallen short. For example, Gavestinel ultimately failed in stroke trials [250]. Encouragingly, newer therapies, like laquinimod, show immunomodulatory benefits in multiple sclerosis [251]. These findings highlight the difficulty of balancing selectivity and polypharmacology. L-701,324, for instance, exhibits strong NMDA receptor antagonism yet also causes unwanted glutamatergic side effects (Table 3) [252]. Future strategies emphasize BBB-penetrant analogs and multi-target ligands exemplified by tasquinimod, including HDAC (histone deacetylase) 4 inhibition and aryl hydrocarbon receptor modulation.

### 4.1. Rational Design Successes and Failure

Refining quinoline scaffolds through targeted synthetic modifications has proven effective in improving receptor selectivity and pharmacokinetic properties [293]. In the case of 7-CKA, introducing a chlorine atom at the 7-position strengthens antagonism at NMDA receptors, surpassing the activity of KYNA [253]. This substitution not only stabilizes the molecule’s conformation at the binding site but also alters its metabolic profile, with potential implications for its clearance and half-life [294]. Gavestinel presents a related example, where the incorporation of sulfonamide groups enhances solubility and may support passage across the BBB, features that are critical for central nervous system activity [295]. While such modifications can improve receptor engagement and bioavailability, they also introduce challenges [296]. Altering chemical structures to enhance efficacy may inadvertently affect safety, particularly as compounds advance toward clinical evaluations [297]. Balancing these factors requires careful attention to the relationship between functional groups and biological outcomes [298]. Through studies of compounds, like 7-CKA and Gavestinel, medicinal chemistry continues to identify structural features that support NMDA receptor modulation, offering a foundation for the development of new treatments targeting neurological disorders.

A clear pattern emerges when considering how discrete structural modifications influence the pharmacological profiles of quinoline-based derivatives. Introducing halogens (e.g., Cl, F) at critical ring positions can improve receptor blockade—especially at NMDA’s glycine site—while often enhancing lipophilicity for better BBB penetration, as seen with 7-CKA. Conversely, sulfonamide substitutions (such as those in Gavestinel) significantly boost solubility yet require careful balancing to avoid off-target interactions. Fluorinated derivatives likewise tend to display enhanced metabolic stability, extending the half-life without substantially raising the toxicity risk. By systematically mapping how each substituent alters binding affinity, membrane permeability, and metabolic clearance, we gain a cohesive SAR framework. Such an approach clarifies why certain motifs are selected to reduce excitotoxicity in neurodegenerative models, enhance anti-inflammatory effects, or leverage immunomodulatory mechanisms for oncology applications. Centralizing these SAR principles not only consolidates the scattered findings but also serves as a blueprint for future chemical refinements and targeted therapeutic design (Table 4).

The challenges of translating quinoline-derived compounds into clinical success are illustrated by the case of Gavestinel. Developed to limit excitotoxic injury following stroke through NMDA receptor antagonism, Gavestinel advanced from preclinical studies with strong supporting data [299]. Yet, in clinical trials, it failed to produce meaningful improvements in patient outcomes, raising questions about the limitations of commonly used experimental models [300]. Stroke in humans presents variability in its onset, severity, and timing that is difficult to replicate in animal studies [301]. Many preclinical trials rely on designs lacking adequate randomization, blinding, or statistical power, factors known to overestimate efficacy in stroke research [302]. Differences in treatment windows also complicate translation [303]. While preclinical models often apply interventions early, real-world scenarios involve delays, with irreversible damage frequently present before therapy begins [303]. Addressing these discrepancies requires trial designs that account for the complexity of human stroke and consider combining neuroprotective strategies with reperfusion approaches [304]. Without such adjustments, promising compounds remain at risk of falling short in clinical settings, regardless of structural refinement or the pharmacological rationale.

The failure of IDO inhibitors in oncology trials has raised important considerations for the development of quinoline-based therapies [305]. Epacadostat, an IDO inhibitor evaluated for melanoma, did not meet its clinical endpoints, partly due to limited patient stratification and the absence of reliable biomarkers to predict treatment responses [306]. Similar issues have been observed with Gavestinel in stroke studies, where variability in disease onset and delayed intervention may have obscured the potential benefits [307]. These examples point to the value of using biomarkers—such as measures of KYN pathway activity or inflammatory cytokines—to guide patient selection and improve trial outcomes [308]. Combining KYNA analogs with immune checkpoint inhibitors or metabolic regulators may also help counteract compensatory mechanisms that reduce therapeutic effectiveness [309]. Trial designs that include real-time pharmacokinetic assessments, like cerebrospinal fluid analysis to verify central nervous system exposure, could support better decision-making during clinical evaluation [310]. Failures, such as epacadostat’s, also reflect the limitations of animal models, which often fall short of replicating the complexity of human pathophysiology [311,312]. Integrating techniques, like metabolomics and single-cell RNA sequencing, into preclinical research may help identify specific patient subgroups who are more likely to benefit from targeted quinoline derivatives.

Laquinimod, a quinoline-3-carboxamide derivative, offers a contrasting example of how molecular design can align with the disease context to support therapeutic development [313]. In multiple sclerosis, where treatment focuses on managing chronic inflammation leading to neurodegeneration rather than responding to acute injury, laquinimod’s ability to reduce pro-inflammatory cytokines and support neuronal preservation has led to more favorable outcomes compared to agents like Gavestinel [314]. Unlike stroke, where delays in intervention often limit the impact of neuroprotective strategies, the progressive nature of multiple sclerosis provides a broader window in which immunomodulation may alter disease progression [315]. These differences raise an important question: how can molecular targets be matched more effectively to the underlying biology of a condition? Laquinimod’s selective focus on immune pathways contrasts sharply with the NMDA receptor antagonism of Gavestinel, highlighting the value of mechanisms that reflect the complexity of the disease environment. While setbacks in clinical translation are common, cases like laquinimod suggest that carefully tailored approaches, applied to appropriate patient groups, may improve the likelihood of success and offer meaningful advances in treating neurodegenerative disorders [316,317].

The varied structural modifications outlined (e.g., halogenation in 7-CKA, sulfonamide substitution in Gavestinel, or the esterification approaches in KYNA analogues) reveal how each tweak can be purposefully directed toward distinct pathologies. For neurodegenerative disorders, strengthening NMDA receptor antagonism while preserving BBB penetrability is pivotal to safeguarding neurons against excitotoxicity. In oncology, motifs that improve solubility and selectivity could be paired with known immune checkpoint inhibitors, leveraging the immunomodulatory potential of quinoline scaffolds. Meanwhile, the anti-inflammatory efficacy hinges on substituents that curb cytokine release without undermining systemic safety. By systematically mapping these structural “levers” to disease-specific objectives, researchers can shape more refined lead compounds and rapidly iterate toward clinically viable designs.

### 4.2. Mechanistic Trade-Offs

L-701,324 has drawn attention for its strong ability to block NMDA receptors and suppress excitatory neurotransmission [318]. In conditions, such as epilepsy and neuropathic pain, this level of inhibition may offer therapeutic benefits [319]. Yet, the same mechanism that provides relief can also carry significant risks [320]. Cognitive disturbances, sedation, and symptoms resembling psychosis have been linked to excessive glutamatergic suppression, raising difficult questions about how far receptor blockade should go [321]. Should drug development focus on narrowing selectivity to avoid unintended effects, or might a broader range of activity offer advantages in disorders shaped by multiple interacting pathways? While some support the latter, NMDA receptor antagonists present clear limitations [322]. Even small deviations in targeting can disrupt neural functions, underscoring the importance of precise molecular engagement [323]. The case of L-701,324 highlights this tension between therapeutic potential and safety [324]. Adjusting the receptor affinity without tipping the balance too far remains a central challenge [322]. As research moves forward, designing compounds that maintain effective modulation while minimizing disruption will be key to expanding the use of NMDA antagonists in clinical settings.

A growing strategy involves developing BBB-penetrant derivatives that build on the neuroprotective potential of KYNA while addressing its limited access to the central nervous system [56]. One example includes modifying KYNA with tert-butyl ester groups, which may improve its transport across the BBB before enzymatic processes release active KYNA within neural tissue [325]. Alongside these efforts, multi-target approaches are attracting interest, particularly for conditions shaped by overlapping biological pathways [326]. Tasquinimod offers a case in point, combining HDAC4 inhibition with aryl hydrocarbon receptor modulation to influence both immune responses and cellular growth [327]. Such dual-action strategies aim to address the complexity of disorders like cancer and autoimmune diseases, where inflammation, proliferation, and tissue damage converge [328]. Whether the goal is to strengthen the delivery of KYNA analogs or to design compounds that coordinate multiple mechanisms, the challenge lies in maintaining therapeutic benefits while controlling off-target effects [329]. Progress will depend on refining these scaffolds through systematic screening, predictive modeling, and careful pharmacokinetic evaluations [330]. Together with biomarker-informed clinical studies, these efforts may guide the development of quinoline-based therapies for neurological, oncological, and inflammatory conditions.

Quinoline derivatives are gaining attention for their ability to influence epigenetic mechanisms, particularly through the modulation of histone deacetylation and DNA methylation [331]. Tasquinimod, targeting HDAC4, has demonstrated potential in addressing both neurodegenerative conditions and cancer, while newer compounds are being designed to expand activity across additional HDAC and DNMT targets [332]. These effects may help to restore tumor-suppressor functions or regulate inflammatory pathways [333]. Genetic variation within the KYN pathway, including polymorphisms in IDOs and KMO, adds another layer of complexity by shifting the balance between protective and harmful metabolites [334]. Combining genotyping with metabolic profiling may guide treatment strategies [335]. Moving forward, refining these molecules will require careful structure–activity studies and genotype-based clinical trials.

Recent findings also underscore the potential of lipid-based nanoparticle systems to improve the delivery of quinoline derivatives across the BBB [336]. For instance, encapsulating these molecules in lipid vesicles can mitigate their rapid clearance, thereby increasing CNS bioavailability [337]. This strategy not only allows for sustained drug release but also reduces off-target toxicity—advances that could reinvigorate clinical interest in repurposing or refining existing quinoline scaffolds [338]. In parallel, polymeric nanoparticles and biomimetic vesicles, such as exosome-based carriers, are emerging as next-generation vehicles for transporting hydrophilic or ionizable quinoline species [339]. By tailoring the particle size, surface charge, and release kinetics, researchers can customize how these agents distribute and act in vulnerable CNS regions. Nanotechnology offers promising avenues to overcome the delivery challenges of polar quinoline derivatives, like KYNA. By encapsulating these molecules in exosomes or polymeric nanoparticles, it is possible to enhance their stability, improve BBB penetration, and achieve targeted release at the site of action. This advanced delivery strategy not only increases the local drug concentration but also minimizes systemic exposure, paving the way for more effective neuroprotective therapies. Incorporating such advanced delivery mechanisms will be crucial for translating preclinical ‘hits’ into viable, patient-friendly therapeutics.

## 5. Next-Generation Kynurenic Acid (KYNA) Analogues: The SZR Series

Structure–activity relationship (SAR) studies yield SZR derivatives with improved pharmacological profiles [340]. SZR-72 shows enhanced neuroprotection, effective blood–brain barrier (BBB) penetration, and behavioral modulation [341,342,343]. SZR-104 displays high BBB permeability and neuroprotective effects in sepsis models [54,344]. SZRG-21 demonstrates the effect on motor activity and emotional behavior [343]. SZR-109 stands out for its strong BBB penetration, suppression of TNF-α, upregulation of TSG-6, and anti-convulsant activity [56,345,346]. While SZR-73 enhances mitochondrial functions and reduces systemic inflammation in sepsis, it does not restore microcirculation. This limitation curtails its overall therapeutic potential [229]. SZR-105, while highly BBB-permeable and anti-inflammatory, requires further validation for its long-term safety [56,345]. Prioritizing SZR-81 may be premature due to poor BBB penetration, although its antidepressant-like effects and neuroprotective potential are promising (Table 5) [347]. Lead optimization may benefit from synergistic strategies. For instance, combining these analogs with IDO inhibitors could more effectively modulate the KYN pathway and boost therapeutic efficacy.

### 5.1. SAR-Driven Innovations

In-depth SAR investigations reveal how subtle modifications to the KYNA core can profoundly affect receptor affinity and metabolic traits [349]. SZR-72, for instance, features a small methyl group that contributes to its enhanced neuroprotective effects, blood–brain barrier penetration, and behavioral modulation—suggesting that subtle steric modifications may play a significant role in optimizing CNS-targeted activity [343]. Meanwhile, SZR-104’s C3 addition of the polar ring system demonstrates how introducing electronegative groups can enhance BBB permeability and support neuroprotection, especially in systemic inflammatory conditions [343]. By tweaking these chemical features—adding a methyl here, attaching a ring system there—researchers can systematically fine-tune key pharmacological properties, like the potency and half-life [350]. Such directed alterations reflect a deeper understanding of how receptor–ligand interactions depend on precise spatial and electronic configurations. In some cases, these modifications not only boost the BBB permeability but also reduce off-target binding, mitigating potential side effects [39]. Additionally, selecting the right functional group can influence polarity and solubility, traits that shape oral bioavailability [351]. Together, these iterative design strategies exemplify the power of SAR-driven innovations to optimize molecules for both efficacy and safety [352]. Beyond SZR-72 and SZR-104, further explorations could unleash even more advanced analogues that are fine-tuned to specific therapeutic goals. As we learn more, SAR mapping remains a cornerstone of rational drug discovery for KYNA derivatives.

Among the ever-growing list of KYNA analogues, SZRG-21 and SZR-109 stand out for their distinct therapeutic profiles in vivo [343,346]. Meanwhile, SZR-72, SZR-104, and SZRG-21 influence motor-associated curiosity and emotional behavior, implying broader neuromodulatory effects [343]. On the other hand, SZR-109 exhibits potent neuroprotective properties, notably suppressing TNF-α production and upregulating TSG-6 [345]. Its ability to effectively penetrate the blood–brain barrier and its anti-convulsant effects—preventing seizure-like activity—further highlight its therapeutic potential in central nervous system disorders [56,346]. The contrasting profiles of these analogues underscore how strategic SAR modifications can yield compounds tailored to specific pathophysiologies. While SZRG-21 appears suited to modulate behavioral aspects, SZR-109 is structurally optimized for neuroprotection [343,346]. These findings emphasize the flexibility of the KYNA scaffold, which allows for fine-tuned substituents to address diverse pathological targets. Further studies on their binding affinities and pharmacokinetics may unlock new therapeutic applications and refine their clinical relevance. Ultimately, SZRG-21 and SZR-109 exemplify how SAR-guided innovation can push the boundaries of KYNA-based therapeutics.

### 5.2. Translational Barriers

Despite ongoing refinements to KYNA analogues, multiple translational barriers must be addressed before these compounds can achieve widespread clinical utility. Prodrug strategies or nanoparticle delivery systems could potentially mitigate such clearance problems, yet these approaches introduce their own complexities in formulation and manufacturing [353,354]. On another front, SZR-105 demonstrates considerable promise but poses its own safety concern: off-target kinase inhibition [56,355]. Even minor structural tweaks can inadvertently shift binding toward other enzymes or receptors, jeopardizing patient safety [343,356].

Recent advances in nanoparticle encapsulation, lipid-based vesicles, and exosome carriers have demonstrated notable improvements in delivering quinoline derivatives across the blood–brain barrier. For example, encapsulating polar KYNA analogs in liposomes can maintain effective drug concentrations in CNS tissues, while minimizing systemic exposure and associated side effects. Complementary to these delivery strategies, computational modeling—particularly machine-learning-driven structure-activity predictions—can highlight potential off-target receptors or enzymes early in the design process. This approach helps to refine scaffolds before in vivo testing, thereby cutting down on costly late-stage failures and patient safety risks. Integrating both cutting-edge formulation methods and in silico screening thus represents a powerful path to overcoming longstanding challenges in quinoline-based neurotherapeutics.

Balancing pharmacological potency with a clean off-target profile is therefore a central challenge in modern drug development [357,358]. Medicinal chemists must undertake detailed SAR investigations, systematically exploring substituents and functional groups to minimize toxicity while preserving therapeutic efficacy [359]. Such iterative optimization demands close collaboration among chemists, pharmacologists, and toxicologists, each focusing on a different aspect of a molecule’s life cycle [360]. By carefully addressing problems, like rapid clearance and unintended kinase inhibition, researchers can pave the way for safer, longer-lasting KYNA-based treatments that ultimately improve patient outcomes.

### 5.3. Path to Lead Optimization

Achieving an optimal lead among KYNA analogues calls for balancing efficacy, selectivity, and favorable pharmacokinetics [361]. Balanced profiles are critical in translational research, where the nuances of absorption, distribution, metabolism, and excretion (ADME) intersect with complex biological pathways [362]. High bioavailability ensures that the compound can be administered conveniently, improving patient compliance and potentially reducing the dosing frequency [363]. Meanwhile, precise receptor selectivity helps to mitigate adverse effects by limiting unwanted off-target binding [364]. These attributes underscore the importance of methodical SAR evaluations, wherein subtle modifications to the parent scaffold can bolster both pharmacodynamics and pharmacokinetics [365]. Complementary techniques, like computational modeling and in vitro assays, further expedite the refinement process, shedding light on metabolic pathways and guiding structural tweaks [366]. By identifying leads in the SZR series, researchers can streamline development cycles and reduce the risk of late-stage failures in clinical trials [367]. Ultimately, focusing on candidates that marry oral bioavailability with selective receptor engagement represents a viable strategy to usher KYNA-based therapies toward their full therapeutic potential.

Adopting combinatorial approaches that integrate multiple therapeutic strategies can heighten the efficacy of KYNA-based treatments [368]. One promising avenue involves combining SZR analogs, designed to selectively modulate GPR35 or NMDA receptors, with established IDO inhibitors that curtail the overall flux through the KYN pathway [369]. Recent findings underscore the intestinal microbiome’s pivotal role in generating, modifying, and regulating KYN pathway metabolites. Microbial enzymes can convert dietary tryptophan into an array of quinoline derivatives that enter systemic circulation, affecting immune homeostasis and neural functions. This dynamic also presents new opportunities for the oral delivery of quinoline-based drugs. Formulations designed to remain stable in the gastrointestinal tract—or even to be activated by specific microbial enzymes—may boost the local or systemic availability of therapeutic agents. Understanding the interplay between the gut microbiota and the KYN route thus represents a crucial frontier for optimizing both the efficacy and safety of next-generation quinoline therapies.

By partially blocking the production of downstream metabolites, IDO inhibitors reduce the burden of neurotoxic or immunomodulatory intermediates, thus creating a biochemical context where KYNA analogs may exert stronger protective or immunoregulatory effects [370]. This two-pronged tactic could help maintain stable levels of beneficial metabolites, like KYNA, while simultaneously minimizing harmful byproducts, leading to more robust interventions in neurodegenerative or inflammatory conditions [371]. Preclinical data suggest that the synergy may stem from the interplay between decreased KYN production and amplified receptor engagement by the analogs [372]. However, identifying optimal drug combinations will require meticulous dose–response studies and toxicological evaluations [373]. In particular, attention to pharmacokinetic profiles, such as how these agents interact or compete for metabolic pathways, is essential to avoid unintended consequences [374]. Ultimately, harnessing a combinatorial approach, like SZR analogs plus IDO inhibitors, illustrates the potential of integrated interventions targeting multiple points along the KYN pathway [375]. Such strategies may yield tangible clinical outcomes that surpass those achievable with single-agent therapies alone.

## 6. Discussion

The KYN metabolic system serves as a critical nexus in metabolism, intricately linking Trp catabolism to the production of neuroactive and immunomodulatory quinoline-based compounds [376]. Central to this pathway are endogenous metabolites, such as KYNA and QUIN, which exert intrinsically distinct and often opposing effects both individually and in relation to each other, modulating the equilibrium between neuroprotection and excitotoxicity, immune responses, and the redox balance [8,377]. Structural nuances within their quinoline scaffolds, including carboxylation, halogenation, and hydroxylation, profoundly influence their receptor specificity (e.g., NMDA, GPR35) and metabolic stability, underscoring the interplay between chemistry and physiology [124]. Despite their therapeutic promise, translational hurdles, such as poor bioavailability, rapid clearance, and paradoxical biological effects, impede clinical progress [378]. This review systematically evaluates endogenous and synthetic quinoline derivatives, emphasizing their structural diversity, mechanistic trade-offs, and therapeutic potential across neurodegenerative, inflammatory, and oncological contexts. By synthesizing structure–activity relationships and translational challenges, we aim to identify a lead compound inspired by KYNA, prioritizing innovations, like prodrug strategies and multi-target engagement, to bridge the gap between preclinical promise and clinical efficacy [379,380]. Our findings aim to catalyze rational drug design for quinoline-based therapies in complex diseases.

Building on the preceding sections, we highlight how research on the chemical identity, structural nuances, and biological roles of quinoline-based metabolites points to significant therapeutic opportunities [225]. Across various endogenous and synthetic derivatives, the document emphasizes the delicate interplay between neuroprotective and neurotoxic effects, demonstrating how minor structural shifts can dramatically influence receptor binding, solubility, and metabolic stability [381]. By exploring molecules, like KYNA and QUIN, alongside analogues engineered for improved pharmacokinetics or selectivity, the synthesis showcases promising targets for conditions spanning neurodegenerative disorders and cancer [382]. Of particular note is the attention placed on bridging preclinical findings with translational barriers, including bioavailability challenges and safety concerns [383]. Overall, this review demonstrates that tuning the quinoline scaffold holds vast potential for advancing novel interventions, underscoring the need for continued interdisciplinary efforts to harness these insights for tangible patient benefits.

These four pillars provide a cohesive blueprint for taking KYNA-inspired quinoline derivatives from the lab to real-world treatments (Table 6). First, mapping disease pathways ensures that each structural motif addresses specific pathogenic processes—be it glutamate excitotoxicity in Alzheimer’s disease or immune dysregulation in rheumatoid arthritis. Next, meticulous SAR refinements guide rational changes in polarity, substituent placement, or prodrug modifications, thereby enhancing target engagement while curbing off-target interactions. Validating each candidate in context-specific models (like transgenic mouse lines for Parkinson’s or tumor xenografts) then confirms that the observed in vitro benefits actually translate into meaningful in vivo outcomes. Finally, integrating biomarker-based selection ensures that researchers can pinpoint which patient subgroups are most likely to benefit, ultimately increasing trial success rates and fostering more precise, personalized therapy. By weaving together these pillars, investigators build a robust framework for innovation—one that promotes both scientific rigor and practical translational impact in the development of next-generation quinoline-based interventions.

Quinoline–metal complexes, particularly 8-hydroxyquinoline chelators, play a crucial role in mitigating oxidative stress in Alzheimer’s disease [133,384,385]. By binding excess metal ions, such as copper and zinc, these compounds prevent the metal-catalyzed production of reactive oxygen species, thereby reducing neuroinflammation and amyloid aggregation [386]. Additionally, their ability to regulate the redox balance and modulate autophagy pathways highlights their potential as multi-target neuroprotective agents [387]. Notably, recent breakthroughs in migraine therapeutics have underscored how targeting neuropeptides, such as calcitonin-gene-related peptide, pituitary adenylate cyclase-activating polypeptide, and vasoactive intestinal polypeptide, can reshape our understanding of neuroinflammation and pain transmission, further emphasizing the need to develop quinoline-based agents with pleiotropic mechanisms [388]. Future research should focus on optimizing their stability and brain permeability to enhance clinical applicability. Structural modifications of KYNA derivatives extend beyond receptor binding, influencing critical downstream pathways, such as mTOR and autophagy [389]. Minor alterations in functional groups can modulate phosphorylation events within the mTOR axis, potentially affecting neuronal survival and inflammatory responses [390]. Additionally, shifts in KYNA’s metabolic profile may alter autophagy-related signaling, impacting cellular homeostasis under oxidative stress conditions [391]. These mechanistic insights highlight the need for targeted modifications that optimize KYNA analogs for neuroprotective or immunomodulatory applications. Such synergy underlines the manuscript’s contribution to research.

Although the KYN pathway is often discussed in terms of glutamate receptor modulation, its complex interaction with serotonin metabolism and mitochondrial function deserves greater attention [23,392]. In particular, QUIN—a downstream metabolite—can significantly impact neuronal energy homeostasis by depleting NAD+ through the de novo salvage pathway [393]. When QUIN accumulates, it not only drives excitotoxic damage but also impairs mitochondrial electron transport, exacerbating oxidative stress and triggering deficits in ATP production [394]. This metabolic interplay may be especially critical in neurodegenerative disorders, where chronic inflammation and mitochondrial dysfunction intersect to accelerate disease progression [395]. Meanwhile, upstream metabolites within the KYN pathway compete for Trp, potentially altering serotonin synthesis and influencing mood regulation [392]. Such biochemical crosstalk helps to explain why patients with conditions like Parkinson’s disease or Alzheimer’s often present with both cognitive and affective symptoms [396,397]. By illuminating how QUIN’s NAD+-depleting properties converge with disrupted serotonin metabolism, researchers can identify new multi-target strategies—such as modulating key enzymes in each branch of Trp degradation. Ultimately, a deeper appreciation of these interconnected metabolic nodes can refine our understanding of disease mechanisms, paving the way for therapeutic approaches that tackle excitotoxicity, energy deficits, and mood dysregulation in a more holistic manner [398,399].

Beyond exploring prodrug designs and multi-target ligands, integrating emerging technologies—particularly CRISPR-based screening—could elevate the originality and impact of quinoline-focused research [400,401]. CRISPR knockouts or knock-ins enable the precise genetic manipulation of targets implicated in the KYN pathway, thereby uncovering how specific enzymes, transporters, or receptors influence the efficacy of quinoline derivatives [402]. This level of control helps to delineate off-target effects and refine SAR predictions, ensuring that lead compounds undergo more robust validation before advancing to clinical trials [403]. Additionally, CRISPR-based functional genomics can be paired with advanced phenotypic assays (e.g., high-content imaging or single-cell transcriptomics) to capture subtle changes in cell viability, immune signaling, or neuronal stress responses [404]. Such multimodal approaches can reveal novel synergy points—for instance, identifying an unanticipated metabolic bottleneck that amplifies or diminishes the therapeutic potency of KYNA analogues [405]. By validating these genetic interactions early in the drug-development pipeline, investigators can better prioritize candidates with the highest translational potential, thereby reducing costly late-stage failures [404]. Ultimately, the marriage of CRISPR technologies and quinoline-focused medicinal chemistry stands poised to yield a new generation of targeted interventions, leveraging enhanced mechanistic precision to tackle neurodegenerative, oncological, and inflammatory pathologies in a truly innovative manner.

The chemical identity extends well beyond a molecule’s basic formula or nomenclature [406]. Subtle shifts in substituents—such as halogens, hydroxyls, or carboxyl groups—can drastically change a quinoline compound’s lipophilicity, stability, and affinity for target receptors [407]. For instance, introducing a chlorine atom at a strategic position may improve BBB penetration, while adding a bulky moiety might decrease solubility or alter enzyme-mediated metabolism [408]. These seemingly minor alterations can pave the way for novel pharmacological profiles, sometimes swinging the balance between neuroprotection and toxicity [409]. Historically, researchers have leveraged rational design methods to develop promising quinoline analogues with heightened specificity, boosted potency, and reduced side effects [245]. Nonetheless, large-scale synthesis remains challenging when certain derivatives exhibit poor yields or require stringent reaction conditions, thus complicating purification steps [410]. Adding to these hurdles, positional isomerism in the fused ring system can generate multiple byproducts that confound analytical assessments and limit overall compound purity [411]. Recognizing such synthetic barriers is critical for streamlining production pipelines and ensuring that lead candidates meet rigorous quality standards [412]. Overall, mapping out how even the smallest functional group can shift a compound’s fate is key to optimizing existing scaffolds and propelling more targeted therapeutic discoveries.

Biological mechanisms rarely operate in isolation. When a derivative modulates a single receptor or enzyme, it often triggers a ripple effect through multiple pathways, especially in the context of the KYN route [413]. Some compounds, for instance, may tamp down neuroinflammation by directly attenuating excitotoxic signals, only to exert a secondary influence on immune cells by altering cytokine release [414]. Such dual or even multi-pronged interactions underscore the need to see beyond straightforward receptor-binding data [415]. For KYN pathway analogues, identifying exact positions in the metabolic cascade reveals potential consequences for the redox balance and neuronal homeostasis: a slight shift in enzyme activity could tip the scales from protective antioxidant mechanisms to detrimental oxidative stress [416]. Yet, despite these insights, many indirect targets remain elusive. Off-target interactions—whether they involve uncharacterized receptors or ancillary enzymes—can complicate both therapeutic efficacy and safety [417]. More rigorous mapping of these secondary pathways, using both in vitro and in vivo models, could pinpoint previously overlooked nodes that either amplify or mitigate a compound’s effects [418]. Bridging such gaps would open the door for more targeted preclinical evaluations and eventually guide clinical trials toward meaningful outcomes [419]. Ultimately, unraveling these intricate webs of interaction is crucial for fully harnessing the therapeutic potential of quinoline-based metabolites.

Recent research has propelled several quinoline-based metabolites from exploratory benchwork to more tangible therapeutic leads [251]. In vitro assays frequently highlight their ability to modulate neuroprotective pathways, suppress inflammatory signals, or even arrest tumor cell proliferation [121]. Moving to in vivo models, some of these candidates have displayed meaningful behavioral improvements in neurodegenerative disease paradigms or slowed the progression of certain cancers [420]. However, conflicting data occasionally arise, with a compound that shows potent in vitro efficacy falling short in animal studies due to issues like rapid metabolism or unanticipated toxicity [421]. Such discrepancies underscore the complexity of translating molecular promise into robust, clinically relevant benefits [422]. Despite these hurdles, certain agents continue to stand out [423]. For example, derivatives with enhanced BBB penetration appear to be especially compelling for psychiatric or neurodegenerative indications, offering a potential edge over older, less permeable analogues [424]. Moreover, targeted delivery systems—ranging from liposomal encapsulations to prodrug designs—are making inroads by bolstering tissue specificity and minimizing off-target effects [425]. Formulation breakthroughs aside, many programs remain in their early clinical stages, where gaps in ADME data or sporadic toxicity reports can stall progress [426]. Ultimately, bridging these translational gaps will hinge on ongoing refinements in compound design, alongside more systematic preclinical and clinical assessments. Overall, these findings underscore how minor structural modifications in quinoline scaffolds can profoundly influence both protective and pathogenic pathways. Strategic changes to receptor specificity, delivery methods, and enzyme targeting therefore emerge as crucial levers for optimizing their therapeutic applications. A coordinated effort—bridging medicinal chemistry, molecular biology, and clinical research—can propel these compounds toward broader utility in complex disorders.

A vital takeaway is that the design principles underpinning classical quinoline derivatives can enrich the ongoing innovation around KYNA-based therapeutics. Whereas older agents (e.g., quinine and fluoroquinolone antibiotics) illustrate how small substitutions affect targeting and safety profiles, the emergent KYNA analogues reveal new avenues for immunomodulatory and neuroprotective actions. By deliberately blending these two perspectives—one rooted in historical clinical success, the other grounded in advanced pathophysiological insights—we open the door to broader applications that transcend any single therapeutic area. This integrative view not only bridges perceived gaps between “old” and “new” quinolines but also sets a trajectory for the next generation of rationally designed, multifaceted treatments.

Though the therapeutic promise of these quinoline derivatives remains compelling, a series of limitations underscores the need for a cautious and well-strategized development path [250]. Low bioavailability, for instance, often impedes their ability to reach efficacious concentrations at the intended site of action, forcing scientists to explore advanced formulation strategies [421]. Prodrug designs and nanoparticle encapsulations stand out as potential solutions, yet they introduce new complexities in manufacturing and regulatory approvals [12]. Additionally, unresolved safety profiles can stall clinical progress, particularly when a compound influences multiple biological pathways that could spark off-target effects [427]. These concerns highlight the importance of rigorous toxicological screening early in the pipeline. Furthermore, complex synthesis methods may hamper large-scale production, driving up costs and limiting accessibility for broader research [428].

Although many quinoline derivatives exhibit substantial therapeutic promise—from antibiotics to neuroprotective agents—recent reports also underscore concerns about potential organ toxicity, off-target enzyme inhibition, and unpredictable drug–drug interactions. For instance, while sulfonamide-modified scaffolds (such as Gavestinel) enhance solubility and CNS penetration, they can also trigger undesired side effects if not thoroughly screened. Similarly, some halogenated derivatives may inadvertently increase cardiotoxic or hepatotoxic risks. Moving forward, a more systematic approach to toxicity evaluations should accompany all efficacy studies in disease models (e.g., stroke, Parkinson’s, oncology) to ensure that potent therapeutic actions are not overshadowed by damaging toxicities. By integrating in vitro cytotoxicity assays, targeted biomarker analyses, and comprehensive in vivo evaluations, researchers can better align these potent scaffolds with real-world clinical safety standards.

Intellectual property barriers can add another layer of difficulty, restricting collaborative efforts and slowing the pace of innovation [429]. In short, no single fix exists, requiring multifaceted strategies and cross-disciplinary collaboration. Recognizing these challenges is essential for shaping the path forward: unless researchers and stakeholders address these technical, financial, and regulatory constraints, the full clinical impact of quinoline-based therapeutics may remain unrealized. By confronting these obstacles head-on, however, the scientific community stands poised to refine existing designs, explore novel delivery routes, and ultimately translate these promising molecules into real-world treatments.

In light of the diverse roles played by KYN-pathway enzymes, such as IDOs and KMO, incorporating robust biomarker strategies into clinical trial designs becomes essential [430]. This shift aligns with emerging paradigms in mental health diagnostics that emphasize biobehavioral precision over categorical rigidity [431,432]. By measuring baseline gene polymorphisms and enzyme expression levels, clinicians can stratify patients according to their potential responsiveness to quinoline-based agents [433]. This approach mirrors successes in targeted oncology, where genomic profiling matches treatments to precise molecular subtypes [434,435]. In the context of neurological and inflammatory diseases, leveraging a similar paradigm could help avoid the ‘one-size-fits-all’ pitfall, ensuring that individuals most likely to benefit—based on their enzyme activity profiles—are prioritized for quinoline therapies [126]. A growing body of research underscores how biomarker-driven stratification can transform trial outcomes for quinoline therapies. Measuring IDO/KMO activity levels or tracking KYN/QUIN ratios can pinpoint which patients exhibit heightened neuroinflammatory or excitotoxic pathways. Similarly, profiling cytokines (like TNF-α, IL-6) may reveal inflammatory ‘hot spots’ where KYNA-based interventions are most effective. Studies in oncology already illustrate how matching patients to treatments based on genetic markers can significantly enhance response rates. Applying a similar approach to neurodegenerative or autoimmune conditions ensures therapies reach those who stand to benefit most. As such, routine biomarker assessments not only accelerate clinical success but also pave the way for personalized, high-impact interventions. Such enrichment not only boosts the probability of clinical efficacy but also reduces the risk of adverse reactions in patients whose metabolic idiosyncrasies lead to off-target toxicity [436]. As studies of KYN pathway polymorphisms progress, stratification methods should become more refined. This refinement can guide the development of targeted interventions that harness the immunoregulatory and neuroprotective power of quinoline derivatives.

Moreover, polymorphisms in IDOs or KMO can drastically alter flux through the KYN pathway, impacting both the magnitude of neuroprotective metabolites (e.g., KYNA) and the generation of excitotoxic compounds (e.g., QUIN) [437]. This genetic variability underscores why certain patient subgroups respond differently—even within the same clinical trial cohort [438]. Incorporating routine genotyping or enzyme activity assays into trial protocols can clarify which individuals possess ‘favorable’ polymorphisms conducive to enhanced drug efficacy [439]. Conversely, identifying high-risk genotypes might prompt dose adjustments or the addition of secondary agents—like antioxidants or immune modulators—to mitigate potential side effects [440]. By interweaving this biomarker intelligence with standard clinical endpoints, researchers can implement adaptive trial designs that dynamically optimize treatment regimens [441]. This personalized approach stands to accelerate the clinical translation of quinoline-based therapies while reducing attrition rates, effectively bridging the gap between compelling preclinical data and consistent patient outcomes.

Despite this manuscript’s thorough exploration of quinoline-based strategies, a comparative perspective on non-quinoline neuroprotectants—such as indole-based IDO inhibitors or flavonoid antioxidants—could strengthen the rationale for prioritizing quinoline scaffolds [442]. Indole frameworks, while effective at modulating certain enzymes in the KYN pathway, often struggle with limited metabolic stability or suboptimal blood–brain barrier penetration [443]. Likewise, flavonoid antioxidants can offer substantial free-radical scavenging capabilities, yet their broad-spectrum activity and relatively weak target specificity can sometimes lead to off-target effects or insufficient impact on key regulatory nodes [444,445]. In contrast, quinoline cores frequently exhibit a more favorable balance of selective receptor binding and pharmacokinetic versatility, enabling the targeted modulation of both excitotoxic and inflammatory cascades [446]. By systematically juxtaposing these scaffold types, researchers would be better equipped to identify which structural features—such as ring fusion motifs or substituent placement—truly confer an edge in neurodegenerative or inflammatory models [447]. Moreover, head-to-head comparisons could clarify cost-effectiveness, synthesis scalability, and the likelihood of off-target liabilities [448]. Ultimately, a robust assessment of non-quinoline contenders would confirm whether quinolines indeed offer the most promising route to next-generation neuroprotectants, thereby ensuring that future research and development efforts are directed toward the scaffolds most likely to yield significant therapeutic benefits.

Looking ahead, several avenues present themselves for propelling this research toward tangible clinical gains. Novel synthetic strategies hold particular promise, as improving the yield or specificity of key intermediates could streamline large-scale production and expedite trials [449]. Equally intriguing is the potential for combining these quinoline derivatives with other pharmacological agents, such as immunomodulators or antioxidants, to tackle multifaceted diseases like neurodegeneration or cancer [450]. These multi-target regimens could not only boost the therapeutic impact but also mitigate resistance or relapse [451]. High-priority experiments will likely involve both in vivo and ex vivo models to confirm mechanistic pathways, refine optimal dosing, and uncover synergistic effects [452]. Biomarker-driven assessments that correlate molecular actions with patient outcomes will be vital, offering a clearer rationale for translating preclinical successes into human studies [453]. Ultimately, refining structural features while embracing combination strategies provides a balanced roadmap for researchers eager to unlock the full therapeutic potential of this diverse molecular class.

Each discussion subsection effectively acts as a mini-review that weaves together foundational knowledge, underscores clinical significance, and illuminates new directions. By clustering these quinoline-based compounds according to their structural traits or metabolic origins—be they endogenous, synthetic, or analogues—the overarching narrative becomes clearer. Patterns in receptor engagement, bioavailability, and therapeutic windows emerge, helping researchers pinpoint critical design features for improvement [454,455,456]. This bird’s-eye perspective also reveals shared challenges, such as scalability and off-target toxicity, which might impede clinical translation [457]. Yet, these pitfalls can guide smarter modifications in functional groups or combined treatment strategies, driving progress [455]. Ultimately, evaluating these diverse compounds side by side creates a roadmap of what works, what fails, and how the field can advance. The collaborative spirit behind collecting these insights promises to transform individual leads into robust, evidence-based interventions grounded in the science of quinoline-based therapeutics. Harnessing these consolidated findings can chart new approaches for KYNA-inspired strategies. Moving forward, researchers should aim to test these compounds in a broad range of disease models, thoroughly assess their long-term safety, and employ biomarker-guided patient selection to personalize treatments. By merging refined chemical modifications with cutting-edge formulation strategies, it becomes possible to fully exploit the immunomodulatory, neuroprotective, and anticancer promise of this emerging quinoline class.

## 7. Conclusions

The structural complexity of quinoline-based metabolites underpins their remarkable versatility across neurodegenerative, inflammatory, and oncological contexts, thereby making them prime candidates for drug development. This review has shown how carefully tuned substitutions—chlorination, fluorination, and esterification—can bolster receptor affinity, metabolic stability, and bioavailability, as demonstrated by derivatives, such as 7-CKA (enhanced NMDA blockade) and SZR-81 (improved oral absorption and GPR35 selectivity). Nonetheless, ongoing hurdles—limited BBB penetration, off-target effects (such as SZR-105’s kinase inhibition), and rapid clearance (e.g., SZR-73)—show that minor modifications may not fully realize these compounds’ therapeutic potential. Moving beyond basic prodrug strategies, a more integrative approach is warranted—one that simultaneously harnesses advanced computational modeling, combinatorial screening, and emerging research on epigenetic modulators. For instance, coupling KYNA analogues with IDO inhibitors could offer synergistic regulation of the KYN pathway, while multi-target ligands, like tasquinimod, highlight the promise of convergent pharmacological mechanisms. Further, employing novel formulation technologies (e.g., nanoparticle-based delivery) and improved in vivo models can strengthen the translational accuracy, fostering therapies that more closely match human pathophysiology. Ultimately, it is by weaving these interdisciplinary strategies into the existing foundation of SAR insights that the next generation of quinoline-based interventions will realize their broad therapeutic potential.

## 8. Patents

P1500356; PCT/HU2017?000014; EP17759330.8A; US15/082,099: WO 2017149333; A1 2017090. P1500356; PCT/HU2016/050034; US 10,857,236B2 (2020); WO2017021748 (EU).

## Figures and Tables

**Table 1:** Biochemical properties of key kynurenine (KYN) pathway metabolites and nicotinic acid (NA). This table presents the primary physiological roles and biochemical features of these compounds, underscoring their relevance to neurotransmission, immunological regulation, and metabolic pathways.

Compounds	Main Characteristics	Ref.
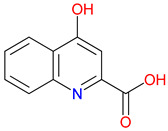 Kynurenic Acid (KYNA)	Neuroprotective—Blocks NMDA and α7nAChR to reduce excitotoxicity KYN pathway metabolite—Modulates neurotransmission and immune response Poor BBB permeability—Limited CNS access, driving prodrug development	[30,51,52] [53,54,55] [56,57,58]
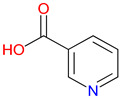 Nicotinic Acid (NA)	Essential vitamin (B3)—Crucial for NAD^+^/NADP^+^ synthesis, supporting cellular metabolism Lipid-lowering effects—Reduces LDL and triglycerides while increasing HDL cholesterol Modulation of nicotinic acid-induced flushing—GPR109A and Langerhans cells drive flushing; DP1 antagonists suppress it	[59,60,61] [62,63,64] [65,66,67]
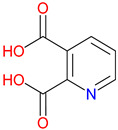 Quinolinic Acid (QUIN)	Excitotoxic agent—Acts as a potent NMDA receptor agonist, leading to neuronal overexcitation and potential cell death Oxidative stress contributor—Promotes free radical generation, exacerbating cellular damage Neurodegenerative implications—Linked to the progression of neurological disorders through its detrimental effects on brain tissue	[68,69,70] [71,72,73] [69,74,75]
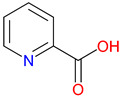 Picolinic Acid	Metal ion chelator—Binds zinc, iron, and other metals, influencing cellular metabolism Immune modulator—Enhances macrophage activity and antimicrobial defense Neuroactive compound—Involved in neurotransmission and potential neuroprotective effects	[76,77,78] [79,80,81] [82,83,84]
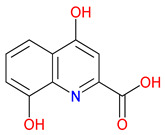 Xanthurenic Acid (XA)	Neuromodulatory effects: Influences neuronal signaling and may modulate receptor activity Putative neuroactive role—Modulates glutamate signaling and may influence neurotransmission Metal chelating properties—Binds with zinc and other metal ions, potentially affecting oxidative stress	[48,85,86] [48,85,86] [87,88,89]
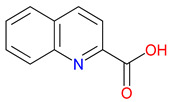 Quinaldic Acid	Quinoline derivative—Structurally related to other KYN pathway metabolites Antimicrobial properties—Exhibits activity against certain bacteria and fungi Potential neuroactive role—May influence neurotransmission, though its biological significance remains underexplored	[90,91,92] [93,94] [92,95]

α7nAChR, α7 nicotinic acetylcholine receptor; BBB, blood–brain barrier; CNS, central nervous system; DP1, prostaglandin D_2_ receptor 1; GPR109A, G protein-coupled receptor 109A; HDL, high-density lipoprotein; LDL, low-density lipoprotein; NAD^+^/NADP^+^, reduced nicotinamide adenine dinucleotide/reduced nicotinamide adenine dinucleotide phosphate; NMDA, N-methyl-D-aspartate.

**Table 2:** Structural and pharmacological overview of quinoline, quinolone derivatives, and pyridinecarboxylic acids. Key compounds within these chemical classes are categorized alongside their main therapeutic actions, biological targets, and significant applications. This table traces the progression from classic antimalarials to contemporary antibiotics, emphasizing how structural changes shape efficacy, safety profiles, and clinical relevance.

Compounds	Main Characteristics	Ref.
Quinoline and quinolone derivatives		
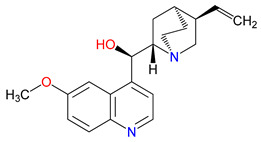 Quinine 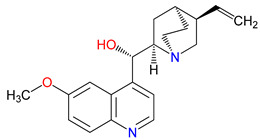 Quinidine	Quinine: Antimalarial agent—Inhibits Plasmodium pp. by interfering with heme detoxification Historically used to reduce fever and pain Muscle relaxant—Helps alleviate nocturnal leg cramps by modulating ion channels Quinidine: Class I antiarrhythmic—Blocks sodium channels, stabilizing cardiac rhythm Chiral isomer of quinine—Shares structural similarities but has distinct pharmacological effects Proarrhythmic risk—Can prolong QT interval ^1^, requiring careful clinical use	[142,143,144] [145,146,147] [148,149] [150,151,152] [153,154,155] [156,157,158]
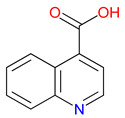 Cinchoninic Acid	Quinoline derivative—Structurally related to quinine and other cinchona alkaloids Metal chelation—Binds with metal ions, potentially influencing enzymatic activity Pharmacological potential—Investigated for antimicrobial and neuroactive properties	[149,159,160] [161,162] [163,164,165]
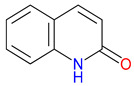 2-Quinolinone 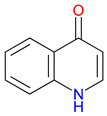 4-Quinolinone	Core scaffold for quinolone antibiotics—Forms the backbone of fluoroquinolones, targeting bacterial DNA gyrase and topoisomerase IV Broad-spectrum antimicrobial activity—Effective against Gram-positive and Gram-negative bacteria Synthetic versatility—Modifiable structure allows for improved potency, pharmacokinetics, and resistance mitigation	[166,167,168] [169,170,171] [170,172,173]
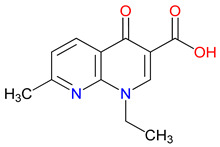 Nalidixic Acid	First-generation quinolone antibiotic—Inhibits DNA gyrase, primarily effective against Gram-negative bacteria Limited spectrum and rapid resistance—Narrow activity and high bacterial resistance limit its clinical use Urinary tract infection treatment—Historically used for urinary tract infections, though largely replaced by newer fluoroquinolones	[174,175,176] [177,178] [179,180,181]
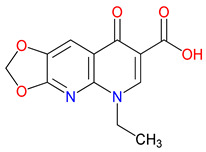 Oxolinic Acid	Early quinolone antibiotic—Inhibits DNA gyrase, effective against Gram-negative bacteria Used in veterinary medicine—Primarily employed for treating bacterial infections in animals Limited clinical use—Replaced by newer fluoroquinolones due to resistance and pharmacokinetic limitations	[182,183,184] [185,186,187] [188]
Pyridinecarboxylic Acids		
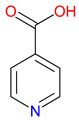 Isonicotinic Acid (INA)	Pyridine carboxylic acid derivative—Structurally related to NA (vitamin B3) Key precursor for isoniazid—Used in the synthesis of isoniazid, a frontline anti-tuberculosis drug Pharmacological potential—Investigated for antimicrobial and metabolic regulatory properties	[189,190] [191,192] [191,193,194]
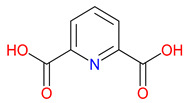 Dipicolinic Acid (DPA)	Metal ion chelator—Strongly binds calcium and other metal ions, playing a role in metal homeostasis Bacterial spore component—Essential for bacterial endospore resistance and heat stability Potential neuroprotective role—Explored for its effects on metal-related oxidative stress in neurodegeneration	[195,196] [197] [198,199,200]
Hydroxy-substituted derivatives		
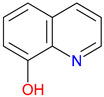 8-Hydroxyquinoline	Metal chelator—Strongly binds iron, copper, and zinc, influencing redox balance and enzymatic activity Antimicrobial and antifungal agent—Exhibits broad-spectrum activity against bacteria and fungi Potential neuroprotective role—Investigated for treating neurodegenerative diseases by regulating metal toxicity	[201,202,203] [204,205,206] [207,208,209]

^1^ QT interval, time between onset of Q wave and end of T wave on electrocardiogram (ECG), reflecting ventricular depolarization and repolarization. NA, nicotinic acid.

**Table 3:** Synthetic exogenous quinoline-based compounds: structures and pharmacological profiles. Synthetic and exogenous quinoline derivatives are outlined here, with an emphasis on their primary mechanisms of action, therapeutic potential, and development hurdles. Each compound is linked to its intended molecular target—from NMDA receptor antagonism to immunomodulatory and anticancer functions—and is accompanied by references for in-depth exploration.

Compounds	Main Characteristics	Ref.
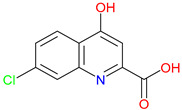 7-Chlorokynurenic Acid (7-CKA)	NMDA receptor antagonist—Blocks the glycine-binding site, reducing excitotoxicity Preclinical behavioral actions—Elicits antidepressant-like effects, blocks NMDA-induced convulsions, and attenuates ischemia-induced learning deficits Phase II clinical trials have demonstrated that 4-chlorokynurenine, a prodrug of 7-CKA, lacks significant antidepressant efficacy in individuals with treatment-resistant depression. It is currently undergoing Phase III evaluation for hypertension and Phase I trials for neurological disorders and neuropathic pain. Additional clinical studies are planned to investigate its potential utility in epilepsy, Huntington’s disease, Parkinson’s disease, various psychiatric conditions, and suicidal ideation.	[253,254] [255,256,257] [125,258]
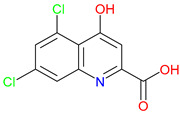 5,7-Dichlorokynurenic Acid	NMDA receptor antagonist—Blocks the glycine site to reduce excitotoxicity Enhanced potency—More effective than KYNA at inhibiting NMDA receptor activity Preclinical behavioral actions—Shows anxiolytic effects and enhances short-term memory and recognition	[259,260,261] [246,259,261,262] [263,264,265]
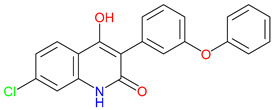 L-701,324	NMDA receptor antagonist—Blocks the glycine site, reducing excitotoxicity Preclinical behavioral evidence—Demonstrates antidepressant-like activity, reduces anxiety-like behavior Potential clinical application–Epilepsy, schizophrenia, and chronic pain	[266,267] [265,268] [269,270,271]
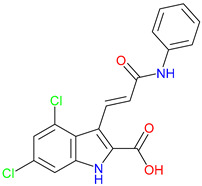 Gavestinel	Glycine site NMDA receptor antagonist—Blocks NMDA receptor activity to reduce excitotoxicity Preclinical behavioral findings—Potential in reducing ischemic damage and modulating certain NMDA-receptor-mediated schizophrenia-like behaviors Stroke neuroprotection candidate—Investigated for acute stroke, primary intracerebral hemorrhage, and acute ischemic stroke, but failed in clinical trials	[272,273] [272,274] [272,275,276]
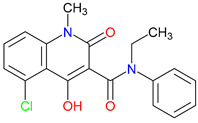 Laquinimod	Immunomodulatory agent—Reduces pro-inflammatory cytokines and modulates immune cell activity Preclinical findings—Improves motor function in a Huntington’s disease model, activates the AhR in the EAE Model of MS Clinical trials—Modestly reduces relapse rates and disability progression and significantly reduces brain volume change atrophy in relapsing–remitting MS, and shows limited efficacy in active non-infectious intermediate, posterior, or panuveitis (NCT02720102)	[277,278,279] [280,281] [282,283,284]
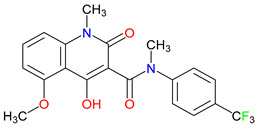 Tasquinimod	Anti-cancer properties—Inhibits tumor angiogenesis, suppresses myeloid-derived suppressor cells, downregulates immune suppressive pathways and inflammatory cytokine signaling S100A9 modulation—Suppresses inflammatory factor expression, potentially inhibist the upregulation of S100A9 in AD, and inhibits MDSC recruitment HDAC4 modulation—potentially influences epigenetic regulation in neurodegenerative or cognitive disorders and potentially controls neuronal memory, plasticity, and learning	[285,286,287] [288,289,290] [291,292]

AD, Alzheimer’s disease; HDAC, histone deacetylase; AhR, aryl hydrocarbon receptor; EAE, experimental autoimmune encephalomyelitis; MS, multiple sclerosis; MDSCs, myeloid-derived duppressor cells.

**Table 4:** Proposed strategies for consolidating and cross-referencing structure–activity relationship (SAR) data.

Recommendation	Approach	Illustrative Examples
Create a Unified SAR Overview	Gather scattered substituent data (chlorine, fluorine, sulfonamides, etc.) in a dedicated section or table.	Summarize each motif (e.g., Cl, F, sulfonamide) and its known effects on receptor affinity or metabolism.
Cross-Reference Key Examples and Motifs	Explicitly reference primary compounds (7-CKA, Gavestinel) to illustrate general rules about halogenation or sulfonamide additions.	For halogens (Cl, F): provide examples showing increased receptor blockade or enhanced lipophilicity (7-CKA, 5,7-DCKA).

SAR, structure–activity relationship; 7-CKA, 7-chlorokynurenic acid; 5,7-DCKA, 5,7-dichlorokynurenic acid.

**Table 5:** Kynurenic acid (KYNA) analogues from the SZR series: structural variations and pharmacological insights. The primary SZR-series analogues derived from KYNA are summarized here, highlighting their structural modifications and core pharmacological properties. The data emphasize their therapeutic relevance to excitotoxicity, neurodegenerative diseases, and related conditions, with references provided for deeper investigation.

Compounds	Main Characteristics	Ref.
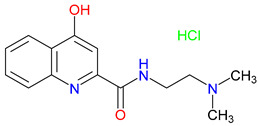 SZR-72	BBB penetration—effectively crosses the blood–brain barrier, enhancing its therapeutic potential in the CNS Neuroprotective effect—Offers enhanced neuroprotection, reduces brainstem c-fos, and induces therapeutic hypothermia Behavioral effects—Alters motor and exploratory behaviors, reducing vertical activity and affecting curiosity-linked emotional and motor responses	[342,343] [341,342] [343]
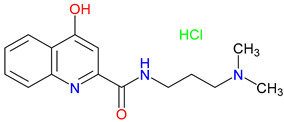 SZR-73	Mitochondrial function enhancement—Enhances mitochondrial respiration and ATP production, improving complex I- and II-linked OXPHOS in a rodent sepsis model Systemic inflammatory activation reduction—Reduces systemic inflammation in sepsis, lowering ET-1, IL-6, and NT levels and XOR activity Microcirculatory effects—Improves mitochondrial function but does not restore microcirculation, unlike KYNA, which improves microvascular perfusion in sepsis	[229] [229] [229]
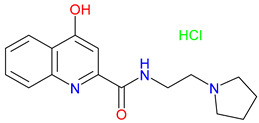 SZR-81	Antidepressant-like effects—Reduces immobility and increasing swimming in FST via serotonin 5-HT system involvement BBB permeability—Poor BBB penetration or metabolic alteration before reaching the CNS Neuroprotective potential–To be explored for use in stroke, neurodegenerative diseases, and inflammatory conditions	[347] [347] [348]
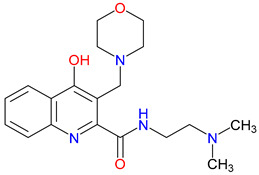 SZR-104	High permeability through the blood—Shows high BBB permeability, surpassing KYNA and analogues Neuroprotective effects in sepsis—Demonstrates neuroprotection in septic rodents, reducing BBB disruption and CNS mitochondrial dysfunction Influence on motor behavior—Increases horizontal exploration, indicating BBB penetration and retention of KYNA-like effects on motor-related curiosity and emotional behavior	[56] [344] [343]
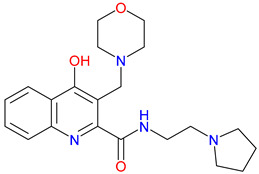 SZR-105	High BBB penetrability—Significantly better BBB permeability than KYNA and earlier analogues due to its dual cationic side chains Potent anti-inflammatory effects—Suppresses TNF-α production and strongly induces TSG-6 Neuroprotective activity in CNS models—Reduces cortical spreading depression propagation in migraine models	[56] [345] [346]
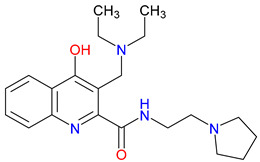 SZR-109	Enhanced BBB penetration—penetrates the BBB more effectively than KYNA Neuroprotective effects—Suppresses pro-inflammatory TNF-α production and upregulates TSG-6 Anti-convulsant activity—prevents or diminishes seizure-like activity in the brain	[56] [345] [346]
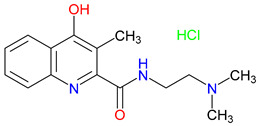 SZRG-21	BBB penetration—Demonstrated efficacy in reducing cytokine levels in colitis models Impact on motor activity—Alters motor activity and exploratory behavior Emotional and behavioral modulation—Affects emotional functions, such as motor-associated curiosity and emotions	[343] [343] [343]

ATP, adenosine triphosphate; BBB, blood–brain barrier; CNS, central nervous system; ET-1; endothelin-1; FST, forced swim test; 5-HT, 5-hydroxytryptamine; IL-6, interleukin 6; KYNA, kynurenic acid; NT, N-terminal peptide; OXPHOS, oxidative phosphorylation; TNF-α, tumor necrosis factor-alpha; TSG-6, tumor necrosis factor-stimulated gene-6; XOR, xanthine oxidoreductase.

**Table 6:** Four pillars for advancing kynurenic acid (KYNA)-based drug design.

Pillar	Primary Objectives	Illustrative Approaches
Pinpoint Disease Pathways	Link structural motifs to specific therapeutic outcomes.	Combat excitotoxicity in Alzheimer’s
Refine SAR Strategies	Optimize structure–activity to boost efficacy and reduce risks.	Use computational docking
Validate in Context-Specific Models	Confirm mechanistic relevance in robust disease models.	Test in transgenic mice (e.g., Parkinson’s)
Incorporate Biomarker-Based Selection	Use metabolic/genomic markers for targeted therapy.	Stratify patients by IDO/KMO polymorphisms

IDO, indoleamine 2,3-dioxygenase; KMO, kynurenine 3-monooxygenase.

## Abbreviations

BBB	blood–brain barrier
7-CKA	7-chlorokynurenic acid
DPA	dipicolinic acid
GPR35	G-protein-coupled receptor 35
HDAC	histone deacetylase
IDOs	indoleamine 2,3-dioxygenases
INA	isonicotinic acid
KMO	kynurenine 3-monooxygenase
KYN	kynurenine
KYNA	kynurenic acid
NA	nicotinic acid
NMDA	N-methyl-D-aspartate
QUIN	quinolinic acid
SAR	structure–activity relationship
Trp	tryptophan
TDO	tryptophan 2,3-dioxygenase
XA	xanthurenic acid
